# The Effect of Genome Parametrization and SNP Marker Subsetting on Genomic Selection in Autotetraploid Alfalfa

**DOI:** 10.3390/genes15040449

**Published:** 2024-04-02

**Authors:** Nelson Nazzicari, Nicolò Franguelli, Barbara Ferrari, Luciano Pecetti, Paolo Annicchiarico

**Affiliations:** Council for Agricultural Research and Economics (CREA), Research Center for Animal Production and Aquaculture, Viale Piacenza 29, 26900 Lodi, Italy

**Keywords:** legumes, alfalfa, genomic selection, SNP calling pipeline, autotetraploidy, polyploidy, genome parametrization, SNP subsetting

## Abstract

Background: Alfalfa, the most economically important forage legume worldwide, features modest genetic progress due to long selection cycles and the extent of the non-additive genetic variance associated with its autotetraploid genome. Methods: To improve the efficiency of genomic selection in alfalfa, we explored the effects of genome parametrization (as tetraploid and diploid dosages, plus allele ratios) and SNP marker subsetting (all available SNPs, only genic regions, and only non-genic regions) on genomic regressions, together with various levels of filtering on reading depth and missing rates. We used genotyping by sequencing-generated data and focused on traits of different genetic complexity, i.e., dry biomass yield in moisture-favorable (FE) and drought stress (SE) environments, leaf size, and the onset of flowering, which were assessed in 143 genotyped plants from a genetically broad European reference population and their phenotyped half-sib progenies. Results: On average, the allele ratio improved the predictive ability compared with other genome parametrizations (+7.9% vs. tetraploid dosage, +12.6% vs. diploid dosage), while using all the SNPs offered an advantage compared with any specific SNP subsetting (+3.7% vs. genic regions, +7.6% vs. non-genic regions). However, when focusing on specific traits, different combinations of genome parametrization and subsetting achieved better performances. We also released *Legpipe2*, an SNP calling pipeline tailored for reduced representation (GBS, RAD) in medium-sized genotyping experiments.

## 1. Introduction

Alfalfa (alias lucerne, or *Medicago sativa* L. subsp. *sativa*) is the most widely grown perennial forage legume in Mediterranean-climate and temperate-climate regions [1]. As a crop, it is prized for its high nutritional content, serving as a vital feed for various livestock species due to its rich protein, vitamin, and mineral composition. As a legume, alfalfa is capable of fixing atmospheric nitrogen through symbiosis with nitrogen-fixing bacteria, thus enhancing soil fertility and diminishing the reliance of growers on synthetic fertilizers in crop rotation systems [2]. Additionally, its deep root system enables alfalfa to access water and nutrients from deeper soil layers, making it resilient in drought conditions and contributing to soil stabilization and erosion control [3].

Unfortunately, alfalfa may appear to be a less attractive choice for growers when compared with other crops, especially cereals. Indeed, progress in alfalfa variety improvement has been slow, owing to a number of concurring factors such as long breeding cycles, low heritability of traits, and a complex genetic structure [1]. More specifically, progress on biomass yield improvement is constrained by a high ratio of non-additive genetic variance due to complementary alleles in the repulsion phase at different loci and intra-locus allelic interactions, as allowed for by autotetraploidy [4]. In practical terms, alfalfa suffers heavily from inbreeding depression and does not allow the creation of pure lines or real hybrids [5]. Since the intrinsic complexiy of the genetic architecture appears irreducible, crop improvement could benefit from advancements in related topics such as sequencing, genomic parametrization, and genomic regression models. Alfalfa genomic selection through models constructed by genotyping parent plants and by phenotyping their half-sib progenies is convenient for synthetic variety development (which can only exploit the additive genetic variance), not only on a theoretical basis [1] but also in view of the relatively greater efficiency of half-sib progeny-based selection relative to other selection schemes for crop yield improvement in this species [6]. Indeed, this genomic selection approach has proved promising for improving biomass yield and key forage quality traits in pioneering studies [7]; however, these used a diploid representation of the tetraploid genome (by pooling the three heterozygous classes of Aaaa, AAaa, and AAAa into a single class) because of largely insufficient SNP reading depth for tetraploid dosage parametrization. 

The development of second- and third-generation sequencing platforms has greatly enhanced the creation of genomic resources for polyploid genomes. Reduced-target next-generation sequencing techniques, such as genotyping-by-sequencing (GBS), have enabled the sequencing of numerous polyploid species and facilitated SNP discovery. Moreover, a variety of software tools and scripts tailored for polyploid crop data analysis has become available. Different SNP calling pipelines for GBS, such as fast-GBS [8], UGbS-Flex [9], and PolyRAD [10], are being utilized in polyploid research [11]. However, no clear a priori indication about the best approach for sequencing and genomic parametrization (i.e., SNP numeric representation) is available. For example, when using a simulated dataset for highly polyploid species, it was found that including allele dosage could improve genomic selection [12]. Other studies instead highlighted a positive effect of allele-ratio parametrization, where the SNPs are just represented as the ratio of observed alleles [13]. Moreover, for practical applications, the cost entailed by an increased reading depth aimed toward tetraploid genome parametrization ought to be justified by a sizable improvement in trait predictive ability compared to a diploid genome representation. The availability of a large number of SNP markers allowed for the effective implementation of genomic selection, by which the phenotyping and genotyping data of a training genetic base (reference population) are combined into a model that estimates breeding values for the target population that will undergo actual selection [7]. Choices on the treatment and representation of genomic data can affect the final performance metric of choice, usually, the predictive ability of the regression. It is, thus, possible to explicitly measure and compare the effect of different strategies, and, consequently, select the optimal combination. In practical terms, the complete set of data (genotypes and phenotypes) is available only for the reference population, while for the target population, only data on genotypes are available. Thus, optimization of the whole regression pipeline is performed on the training population, usually in a cross-validation scheme [14].

The aim of this study was to optimize genomic regressions for alfalfa breeding under the hypothesis that genomic data representation and filtering affect predictive ability. In particular, we investigated the effects of (1) genome parametrization (tetraploid allele dosage vs. diploid vs. observed allele ratios); (2) subsetting SNPs located only in the genic areas, i.e., comparing the effects of keeping markers located only in coding regions of the genome vs. all the available markers; (3) filtering the genomic data based on various thresholds regarding the missing rate and reading depth. The plant material used was from a European reference population obtained by intercrossing several elite semi-dormant cultivars bred in different countries. The material was grown in either a moisture-favorable or a drought-prone environment. Together with the analyses, we release *Legpipe2*, an open-source SNP calling pipeline that guarantees reproducibility.

## 2. Materials and Methods

### 2.1. Plant Material and Phenotyping

The 10 contributing cultivars were selected according to breeders’ indications on best-performing material in seven countries, encompassing more cultivars for countries with larger alfalfa cropping areas. The cultivars were Beatrix, Costanza, and Cuore Verde from Italy, Fado and Galaxie from France, Cezara from Romania, Dara from Bulgaria, Mediana from Serbia, Morava from the Czech Republic, and Vanda from Slovakia, which underwent two generations of intercrossing by bumble bees (*Bombus terrestris*) under insect-proof cages. A set of 143 genotypes was randomly chosen for this study, followed by genotyping them and phenotyping their half-sib progenies in a two-year experiment carried out in Lodi, northern Italy, on a large phenotypic platform. The platform consisted of 6 large (24.0 m × 1.6 m × 0.8 m deep) bottomless containers made of concrete 6 cm thick, filled with local sandy-loam soil, under a rainout shelter provided with a double-rail irrigation boom. Three separate containers represented replicates of a managed environment with imposed severe drought stress, while the other three containersthe replicates for a moisture-favorable managed environment. All containers were separated by alleys 75 cm wide. The irrigation boom above each container was provided with lateral baffles that prevented water from drifting between contiguous containers, which was especially important when they represented different environments. Moreover, the concrete floor of the alleys was paved with a water-proof rubber layer, further preventing undesired leakage and infiltration beneath the containers of any water that dripped onto the alleys. The experiment was established as an α lattice with 16 incomplete blocks of 9 plots each within each replication (a ‘filler’ entry was added to the 143 half-sib families). The area of each plot measured 0.24 m^2^ (0.8 m × 0.3 m) and included 4 rows of 10 plants, each spaced 7.5 cm within and across the rows (plant density = 166.7 m^−2^). The four front plants of the plot were treated as border plants and discarded from the harvest area. The sowing took place in late winter (early March 2022) into plug trays kept in a greenhouse, and seedling transplantation was performed in the platform after eight weeks (early May). Mineral fertilization was incorporated into the seedbed prior to transplantation at the rates of 27 kg ha^−1^ N, 46 kg ha^−1^ P_2_O_5_, and 50 kg ha^−1^ K_2_O. After an initial period of favorable growth, supplying 180 mm of irrigation to all containers, the 2 conditions of water availability were applied, starting from the beginning of July 2022. The two conditions were meant to represent contrasting environments for semi-dormant material across Italy, namely, the rainfed, stressful environment mostly occurring in Central Italy, and the favorable, irrigated environment mainly occurring in the northern part of the Po Valley. During the first year (July–December 2022), the moisture-favorable condition plots received 445 mm of irrigation (in 2 applications per month) while the stressful condition plots received 230 mm of irrigation (in 1 application per month). In the second year (January–December 2023), the irrigation amounts were 825 mm and 375 mm, respectively. The dry biomass yield was recorded on a plot basis in both conditions by hand-clipping all the living plants within the harvest area at a cutting height of 5 cm from the ground, then immediately oven-drying the whole plot biomass at 60 °C for 4 days to a constant weight. Four harvests were made in the first year (between mid-July and late October), and six in the second year (between mid-April and mid-October). However, due to growth impairment caused by the drought stress, only two harvests were made from the stressed treatment plots in the first year (skipping the harvests in August and September), and three in the second year (skipping the harvests in June, July, and early September). We analyzed the total dry matter yield across years, which spanned across 10 harvests for the moisture-favorable condition plots and 5 harvests for the drought-stressed plots since this variable meaningfully represented the crop yield (irrespective of the number of harvests in each condition). The onset of flowering was recorded as the number of days from the day of harvest to the date when open flowers were visible on 10% of the plants per plot. The character was assessed on the regrowth that followed some harvests across the two years (twice in the first year, once in the second year). Leaf size was recorded in early July of the first year, just before the implementation of the two moisture treatments, by measuring the maximum length and width of the central leaflet of a representative leaf (usually, the third or fourth from the uppermost vegetative node) from four random plants per plot and computing the leaf area, expressed in cm^2^, as length × width.

### 2.2. Experimental Design Solution, BLUPs Computation, and Heritability

Broad-sense heritability was estimated according to [15] as the ratio of the genetic variance $\sigma_{g}^{2}$ to the phenotypic variance $\sigma_{p}^{2}$:

(1) $$H^{2}=\frac{\sigma_{g}^{2}}{\sigma_{p}^{2}}$$

where $\sigma_{p}^{2}$ depends on the variance components for genotype $\sigma_{g}^{2}$, experimental error $\sigma_{e}^{2}$ and the number of replicates $n_{r}$, according to the formula:

(2) $$\sigma_{p}^{2}{=\sigma }_{g}^{2}+\frac{\sigma_{e}^{2}}{n_{r}}$$

Best linear unbiased predictions (BLUPs) were then used as phenotypic data for genomic regression [16]. The BLUPs were computed by solving a mixed-model equation where the genotype effect is included as random, i.e., by summing the model intercept (overall mean) to the random effects associated with each genotype, as described in [17]. Heritability and BLUP value computations were carried out using the R-package INTI [18]. For total dry matter measurement, recorded in two environments, we verified the occurrence of half-sib progeny × environment interactions in an analysis of variance including the factor environment, progeny, and replication, and estimated the genetic correlation coefficient for the half-sib progeny response across environments according to the method used in [19].

### 2.3. DNA Extraction, Library Preparation, and Sequencing

Genomic DNA was extracted from the young leaves of each plant using the DNeasy Plant Mini Kit (Qiagen, Milan, Italy). Nucleic acid was quantified by a Quant-iT™ PicoGreen™ dsDNA Assay Kit (P7589, Life Technologies, Milan, Italy), checking its quality by 1% agarose gel electrophoresis. A trial digestion process was carried out on 10% of the DNA samples using the Optizyme EcoRI restriction enzyme (25,000 U, Fisher BioReagents, Rodano, MI, Italy), to compare bands of cut and uncut DNA. The reaction was performed at 37 °C for 1 h and the enzyme was deactivated at 65 °C for 20 min. DNA samples were sent to The Elshire Group Ltd. laboratory (Palmerston North, New Zealand) for outsourced library preparation and sequencing. GBS data were generated according to Elshire et al.’s method [20] with the following changes: we used 100 ng of genomic DNA and 3.6 ng of total adapters and restricted the genomic DNA with the ApeKI enzyme (NEB New England Biolabs, Ipswich, MA, USA, R0643L); then, the library was amplified with Kapa Taq polymerase α (KAPA Library Amplification Readymix, Kapa Biosystems, Wilmington, MA, USA, KK2611) by 14 PCR cycles.

The library was sequenced at the Elshire Group Ltd. facility (Palmerston North, New Zealand) using an Illumina X Ten platform with 150 bp paired-end reads. Each sample was repeated three times on three different lanes. The raw reads were collated before demultiplexing.

The raw reads have been deposited in the NCBI RSA archive under submission number PRJNA1092606.

### 2.4. SNP Calling, Filtering, and Genome Parametrization

SNP calling was executed using the *Legpipe2* pipeline, which is released together with this paper. The configuration file necessary to reproduce the actual SNP calling is available in File S1 in the Supplementary Materials. As the reference genome, we used the sequence obtained from Long et al., 2022 [21], which consists of the full resequencing of each of the four copies of each chromosome. For reads alignment, we selected the longest copy for each chromosome.

The obtained variants were filtered for quality *(phred* score >= 40), minor allele frequency (MAF ≥ 5%), with several levels of missing per marker (5%, 10%, and 20%) and of minimum total reads (10, 20, 30, and 40). The combination of the above filtering produced 12 different genomic datasets, to be further analyzed in parallel.

After SNP calling, the data were transformed in three different genomic parametrizations: allele ratios, tetraploid SNP dosage, and diploid SNP dosage.

Allele ratios are defined as:

AR = *a*/(*A* + *a*) (3)

where *a* is the number of reads containing the alternative allele and *A* is the number of reads containing the standard allele. The allele ratios are, thus, defined in the [0, 1] interval. Allele ratios were computed using a custom R script.

The second genomic parametrization is tetraploid SNP dosage and aims to model the actual number of alternative alleles present at each SNP site. As such, for each SNP sample pair, the final result is an integer number between zero (the homozygote of the same allele found in the reference genome) and four (the homozygote of the alternative genome), with in-between values of between one and three representing the three types of heterozygotes possible in a tetraploid genome. This parametrization was obtained using the *multidog* function from the *updog* R package [22] version 2.1.3, with the parameters *ploidy = 4* and *model = ”norm”*. Once the dosages were obtained, the SNPs were further filtered, discarding the markers with the *bias* parameter outside the [e^−1^, e^1^] range.

The third genomic parametrization is of diploid SNP dosage and is a simplification of the tetraploid SNP dosage obtained, collating the three possible heterozygotes in a single bin of intermediate value between the two homozygotes. In practical terms, each SNP sample pair was represented by an integer value between zero (the homozygote of the reference allele) and two (the homozygote of the alternative allele). Regardless of the dosage, all the possible heterozygotes were represented by a value of one.

SNPs were also qualified as belonging or not belonging to genic regions, using the information available with the reference genome [21]. As such, we compared the regression results using either the full set of SNPs or only the SNPs coming from genic regions, or only the SNPs coming from non-genic regions.

### 2.5. Genomic Regression

The genomic predictions were investigated for all quantitative traits (dry matter in favorable and stressed conditions, onset of flowering, and leaf size) by using a ridge regression BLUP (rrBLUP) [23]. Genotypic data for the genomic regressions were produced to test the effects of different levels of filtering (on the missing rate and number of reads), genome parametrization (allele ratio, tetraploid, and diploid), and SNP selection (all SNPs, genic regions only, and non-genic regions only) for a total of 144 different configurations. For each configuration, the predictive ability of the model was measured as Pearson’s correlation between true and predicted phenotypic values in a 10-fold cross-validation scheme, repeated 10 times (and averaged) for numerical stability using the R package GROAN [24] version 1.3.1.

## 3. Results

### 3.1. Phenotypic Analysis

Table 1 reports broad-sense heritabilities and descriptive statistics for the four focus traits. Genetic variation among half-sib progenies was significant for every trait (*p* < 0.05). The highest heritability was found for the onset of flowering (0.690), followed by leaf size (0.550) and total dry matter in the favorable environment (0.529). The lowest heritability (0.302) was observed for total dry matter in the drought-stressed environment. Due to the plant growth reduction and the lower number of harvests caused by drought stress, the DMY was 6.84 t/ha in the stressed environment compared to 16.50 t/ha in the favorable environment. The presence of stress also reduced the genetic coefficient of variation for total dry matter yield from 9.2% in the favorable environment to 5.5% in the stressed environment. The coefficient of variation for experimental error was almost identical for yield in the two environments.

The genetic correlation for half-sib dry matter yield across the two environments was relatively high, namely, r_g_ = 0.82, despite the occurrence of highly significant half-sib progeny × environment interactions (*p* < 0.001). Total dry matter yield in the favorable environment displayed a modest positive phenotypic correlation with leaf size (r = 0.14) and a negative correlation with the onset of flowering (r = −0.25), whereas the onset of flowering and leaf size correlated positively (r = 0.27).

### 3.2. Sequencing, SNP Calling, and Filtering

Sequencing produced an average of 7.5 Mreads per sample. Table 2 reports the resulting number of SNP markers, depending on the applied filters and on the required number of reads per locus, allowed maximum missing rate, and parametrization. As expected, the total number of SNP markers shrank as the filter parameters became more stringent (higher required reads and lower accepted missing rates), ranging from 2387 to 19,668 markers. The table does not report the number of markers for diploid parametrization since, by construction, they are exactly the same as the tetraploid ones.

Genome parametrization heavily influenced the final number of markers since many markers were rejected during the SNP dosage calling, due to an insufficient number of reads to support proper dosage estimation. This became particularly evident with stricter filtering on the missing rate, e.g., minimum reads per locus = 10 and a maximum missing rate = 5% resulted in about twice as many ratio markers as dosage markers. For comparison, with a maximum missing rate = 20%, the number of resulting markers was about the same as with the two genome parametrizations.

Table 2 also reports the number of SNP markers located in genic regions. While the absolute values changed with filtering and parametrization, the fraction of markers in genic regions was stable, at 73.9% on average.

### 3.3. Genomic Regressions

**Figure 1:**
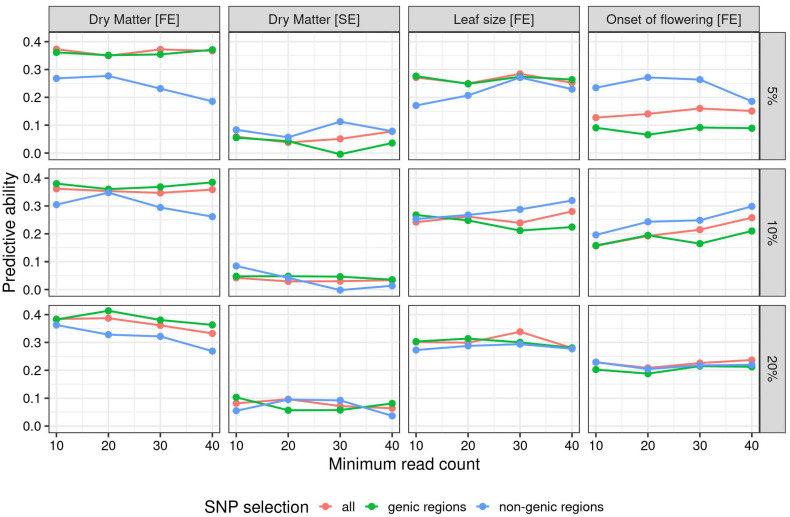
Predictive ability for different levels of minimum reads per locus (*x*-axis), maximum missing rate per locus (rows), SNP selection (line color), and trait (column). SNPs are parametrized as ratios. For tetraploid dosage parametrization, see Supplementary Figure S1.

Genotypic data for genomic regressions were produced to test the effects of different levels of filtering (on missing rate and number of reads), genome parametrization (allele ratio, tetraploid, and diploid), and SNP selection (all SNPs, genic regions only, and non-genic regions only) for a total of 144 different configurations. For each configuration, the predictive ability of the model was measured as Pearson’s correlation between true and predicted phenotypic values in a ten-fold cross-validation scheme. Figure 1 shows the effects on the predictive ability of the choice of filtering (for missing rate and read depth) and SNP subsetting (all markers vs. only genic regions, vs. only non-genic regions). The figure presents the results for SNP ratio parametrization. Figure S1 does the same for the tetraploid parametrization. The full list of results is reported in Table S1.

Figure 1 highlights several trends. The four traits exhibited different patterns, with yield in the favorable environment showing predictive ability values that were always higher than yield in the stressed environment. Leaf size and onset of flowering showed values closer to the yield in the favorable environment, if slightly lower. The use of markers from non-genic regions was heavily penalized in the yield in favorable conditions, where using the marker from genic regions yielded slightly better results than using all markers. For yield in stressed conditions and leaf size, subsetting the markers to genic regions did not appear influential, as shown by the three lines intertwined without a clear advantage. The onset of flowering findings reversed the pattern found concerning yield in favorable conditions, achieving the highest predictive abilities when subsetting the markers from non-genic regions.

Table 3 reports the configuration for each trait giving the highest predictive ability, which ranged from 0.168 (dry matter in the stressed environment) to 0.414 (dry matter in the favorable environment). Three out of four traits maximized the predictive ability when using the more stringent filtering on missing rate, the exception being dry matter in a favorable environment, for which the loosest filtering was best. With regard to genome parametrization, yield in stressed conditions and leaf size achieved the best performances with diploid parametrization, while the onset of flowering was favored by tetraploid parametrization, and yield in favorable conditions by the allele ratio. Regarding SNP selection, two traits (yield in favorable conditions and leaf size) achieved the best results by using SNPs from coding regions, while the other two traits achieved the best results by using SNPs from non-coding regions.

By averaging the predictive ability results over all the tested configurations, it was possible to single out the effect of specific filtering. On average, the allele ratio provided an advantage in terms of average predictive ability (0.232), followed by the tetraploid dosage (0.215) and the diploid dosage (0.206). Averaging over the SNP subsets revealed an advantage using all SNPs (0.226), followed by genic regions (0.218) and then by non-genic regions (0.210).

### 3.4. Released Software: Legpipe2

Together with this study, we are releasing *LegPipe2 version 1.0*, a SNP calling pipeline aimed at reduced representation sequencing (GBS and RAD alike), which is freely available at https://github.com/ne1s0n/legpipe2 (accessed on: 29 March 2024).

The design of *Legpipe2* is inspired by existing pipelines like dDocent [25], with improvements in modularity, log management, and general flexibility. All operations depend on a single configuration file, which can be shared together with the *Legpipe2* version to ensure data reproducibility. Apart from outputting a standard .vcf file [26], *Legpipe2* already contains filtering and data manipulation steps so that the data can easily be imported into other software, e.g., into R/updog [27] version 2.1.3.

Internally, *Legpipe2* uses the GVCF workflow from the GATK/HaplotypeCaller [28] suite, thus ensuring low memory requirements. Other steps are implemented with standard state-of-the-art software, such as Bowtie 2 [29] (for alignment), Picard, and SAMtools [30] (for data manipulation and filtering), and FASTX [31] (for trimming).

## 4. Discussion

Agriculture is a water-intensive activity, and drought exacerbates the competition for water resources, particularly in regions already facing limitations in water availability. Alfalfa is known for its fairly high resilience to drought, but further genetic improvement of its drought tolerance would be needed in several regions because of the changing climate, a process that is challenged, among other reasons, by the difficulty of coping with an autotetraploid genome. In this study, the definitely lower genomic prediction ability for total dry matter yield observed in the drought-prone environment relative to the moisture-favorable one (0.17 vs. 0.41) agrees with earlier findings [7,32]. In particular, for a Mediterranean alfalfa reference population using diploid genome parametrization, a progressive decrease in predictive ability was found across the managed environments, ranging from moisture-favorable (0.35) to moderately stressed (0.26), to heavily stressed (0.03), as well as prediction abilities in the range of 0.12–0.23 for drought-prone agricultural environments. It was previously found [32] that a decreasing predictive ability regarding increasing drought stress was paralleled by a progressive increase in experiment error CV (12.6% vs. 19.5% vs. 30.1%) in the presence of a similar genetic variance. In this study, the same pattern was found for the genetic coefficient of variation, probably because of the smaller variations in drought tolerance that one could expect in European germplasm relative to a Mediterranean reference population. Due to the controlled experimental conditions, the experiment error CV was, however, more stable here, with stressed and favorable conditions showing almost the same values (15.2% and 15.4%, respectively).

Our study provides an unprecedented assessment of the potential advantage of allele dosage imputation for alfalfa genomic predictions. The extent of this advantage varied largely in earlier studies on other species. A study of the perennial autotetraploid forage grass *Panicum maximum* reported a remarkable advantage of allele dosage imputation over the diploid model when pooling the heterozygote classes, with increases in predictive ability of about 50% for leaf dry matter, 42% for crude protein content, and 18% for in vitro digestibility [33]. In contrast, the advantage of allelic dosage imputation was minimal for predicting the agronomic traits of interspecific hybrids of the tropical grass *Urochloa* spp. [34], possibly because of the segmental allotetraploid (partly autotetraploid and partly allotetraploid) genome of this material. In highly polyploid species like sugarcane and sweet potato, it was shown that the inclusion of allele dosage improves genomic prediction only when there is a high frequency of heterozygous genotypes [12]. In contrast, in blueberry, it was found that with low depth sequencing, the use of allele ratio parametrization and simplified diploidization brought results similar to those for full polyploid parametrization [13].

In this study, the disadvantage of a diploid genome representation relative to a tetraploid genome or its approximation as provided by the allele ratio was not large, suggesting that using a diploid representation, as necessarily required when adopting lower sequencing effort, may be convenient for some traits in terms of genomic selection cost efficiency in comparison with more expensive, albeit more informative, genotyping options.

Identifying the optimal combinations of data treatment, filtering, and genome representation may be important to maximize the genome-enabled predictive ability of alfalfa. Our comparison of the allele ratio relative to the tetraploid allele dosage, based on the same sequencing data, suggested that the allele ratio could be the preferred alternative, bringing an average +7.9% increase in predictive ability when compared to tetraploid dosage (+12.6% when compared to diploid). This approach, which is computationally simpler and avoids the problems associated with the misclassification of genotypic classes, produced genomic selection models that were about as accurate as those based on estimated genotype classes in an earlier blueberry study [35]. However, the advantage of the allele ratio held true only on average in our study. In fact, allele ratio parametrization was selected as the best configuration in only one trait out of four. A similar pattern was found when examining the effect of SNP selection. While using all available markers was preferable on average (+3.7% vs. genic regions, +7.6% vs. non-genic regions), that choice was never the best-performing option when looking for the best configuration for each single trait. This finding highlights the layered interactions between data representation, data filtering, and genomic regression performances. From the methodological perspective, we propose, therefore, that genome parametrization and SNP marker selection become part of the options routinely explored when optimizing genomic regression.

## Figures and Tables

**Table 1:** Broad-sense heritabilities, coefficient of genetic variation (CVg, with significance levels of ‘***’ 0.001, ‘**’ 0.01, and ‘*’ 0.05), coefficient of variation for the experimental error (CVe), mean values and value ranges for the four studied traits, measured in either favorable (FE) or stressed (SE) environments.

Trait	Broad-Sense Heritability	CVg (%)	CVe (%)	Mean	Range
Onset of flowering (FE)	0.690	6.2 ***	7.2	20.61	18.24–22.79
Leaf size (FE)	0.550	6.1 ***	13.4	3.31	2.94–3.78
Dry Matter (SE)	0.302	5.5 *	15.2	6.84	6.23–7.34
Dry Matter (FE)	0.529	9.2 ***	15.4	16.50	14.06–19.56

**Table 2:** Number of SNP markers for each combination of filtering on the minimum required number of reads per locus and on the maximum missing rate per locus. Markers are reported as parametrized as tetraploid dosage or as the ratio between alleles. Aside from the total number of markers, the number of markers in genic regions only is also reported, given in absolute values and relative to the total number of markers.

Minimum Reads Per Locus	Maximum Missing Rate per Locus	Dosage SNPs		Ratios SNPs	
		All	Genic (%)	All	Genic (%)
10	5%	5758	4342 (75.41%)	11,965	8771 (73.31%)
10	10%	11,440	8453 (73.89%)	15,422	11,197 (72.6%)
10	20%	17,933	13,088 (72.98%)	19,668	14,058 (71.48%)
20	5%	4162	3147 (75.61%)	7813	5758 (73.7%)
20	10%	8576	6338 (73.9%)	10,660	7791 (73.09%)
20	20%	13,491	9916 (73.5%)	14,243	10,321 (72.46%)
30	5%	3225	2439 (75.63%)	5688	4205 (73.93%)
30	10%	6715	5006 (74.55%)	8021	5919 (73.79%)
30	20%	10,876	8035 (73.88%)	11,306	8251 (72.98%)
40	5%	2387	1814 (75.99%)	4076	3024 (74.19%)
40	10%	5386	4034 (74.9%)	6248	4630 (74.1%)
40	20%	9065	6733 (74.27%)	9278	6822 (73.53%)

**Table 3:** The configuration of SNP filtering and parametrization corresponding to the highest predictive ability is reported for each trait.

Trait	SNP Selection	Parametrization	Maximum Missing Rate per Locus	Minimum Reads per Locus	Predictive Ability
Dry Matter (FE)	coding regions	tetraploid	20%	20	0.414
Dry Matter (SE)	non-coding regions	diploid	5%	30	0.168
Leaf size (FE)	coding regions	diploid	5%	10	0.347
Onset of flowering (FE)	non-coding regions	allele ratio	5%	40	0.301

## Data Availability

Raw reads have been deposited to NCBI RSA archive under submission number PRJNA1092606.

## Supplementary Materials

The following supporting information can be downloaded at: https://www.mdpi.com/article/10.3390/genes15040449/s1, Figure S1. Predictive ability for different levels of minimum reads per locus (*x*-axis), maximum missing rate per locus (rows), SNP selection (line color), and trait (column). SNPs are parametrized as tetraploid dosage. Table S1. Predictive ability for all tested SNP filtering configurations. File S1. Legpipe2 configuration.
