# Supplementary material for: The Effect of Genome Parametrization and SNP Marker Subsetting on Genomic Selection in Autotetraploid Alfalfa
Source: Genes (Basel). 2024 Apr 2;15(4):449. doi: 10.3390/genes15040449 (PMC11050091; doi:10.3390/genes15040449)
Supplement: Supplementary file 1 [file genes-15-00449-s001.zip › genes-2920298-supplementary/Supplementary Figure 1.odt]

Supplementary Figure 1. Predictive ability for different levels of minimum reads per locus (x axis), maximum missing rate per locus (rows), SNP selection (line color) and trait (column). SNPs are parametrized as tetraploid dosage.
