# Supplementary material for: The Effect of Genome Parametrization and SNP Marker Subsetting on Genomic Selection in Autotetraploid Alfalfa
Source: Genes (Basel). 2024 Apr 2;15(4):449. doi: 10.3390/genes15040449 (PMC11050091; doi:10.3390/genes15040449)
Supplement: Supplementary file 1 [file genes-15-00449-s001.zip › genes-2920298-supplementary/Supplementary Table 1.odt]

Supplementary Table 1. Predictive ability for all tested SNP filtering configurations.

| Trait | Minimum reads per locus | Maximum missing rate per locus | SNP parametrization | SNP selection |
| --- | --- | --- | --- | --- |
| All | Genic regions | Non-genic regions |
| Leaf size [FE] | 5% | 10 | tetraploid | 0.27 | 0.28 | 0.17 |
| Leaf size [FE] | 5% | 10 | diploid | 0.29 | 0.35 | 0.15 |
| Leaf size [FE] | 5% | 20 | tetraploid | 0.25 | 0.25 | 0.21 |
| Leaf size [FE] | 5% | 20 | diploid | 0.31 | 0.3 | 0.2 |
| Leaf size [FE] | 5% | 30 | tetraploid | 0.28 | 0.27 | 0.27 |
| Leaf size [FE] | 5% | 30 | diploid | 0.32 | 0.32 | 0.22 |
| Leaf size [FE] | 5% | 40 | tetraploid | 0.25 | 0.26 | 0.23 |
| Leaf size [FE] | 5% | 40 | diploid | 0.3 | 0.33 | 0.22 |
| Leaf size [FE] | 10% | 10 | tetraploid | 0.24 | 0.27 | 0.25 |
| Leaf size [FE] | 10% | 10 | diploid | 0.28 | 0.29 | 0.25 |
| Leaf size [FE] | 10% | 20 | tetraploid | 0.26 | 0.25 | 0.27 |
| Leaf size [FE] | 10% | 20 | diploid | 0.27 | 0.27 | 0.24 |
| Leaf size [FE] | 10% | 30 | tetraploid | 0.24 | 0.21 | 0.29 |
| Leaf size [FE] | 10% | 30 | diploid | 0.29 | 0.28 | 0.29 |
| Leaf size [FE] | 10% | 40 | tetraploid | 0.28 | 0.22 | 0.32 |
| Leaf size [FE] | 10% | 40 | diploid | 0.33 | 0.25 | 0.34 |
| Leaf size [FE] | 20% | 10 | tetraploid | 0.3 | 0.3 | 0.27 |
| Leaf size [FE] | 20% | 10 | diploid | 0.28 | 0.27 | 0.25 |
| Leaf size [FE] | 20% | 20 | tetraploid | 0.3 | 0.31 | 0.29 |
| Leaf size [FE] | 20% | 20 | diploid | 0.32 | 0.32 | 0.28 |
| Leaf size [FE] | 20% | 30 | tetraploid | 0.34 | 0.3 | 0.29 |
| Leaf size [FE] | 20% | 30 | diploid | 0.28 | 0.27 | 0.29 |
| Leaf size [FE] | 20% | 40 | tetraploid | 0.28 | 0.28 | 0.28 |
| Leaf size [FE] | 20% | 40 | diploid | 0.28 | 0.25 | 0.26 |
| Leaf size [FE] | 5% | 10 | allele ratio | 0.29 | 0.29 | 0.29 |
| Leaf size [FE] | 5% | 20 | allele ratio | 0.3 | 0.3 | 0.28 |
| Leaf size [FE] | 5% | 30 | allele ratio | 0.31 | 0.25 | 0.32 |
| Leaf size [FE] | 5% | 40 | allele ratio | 0.32 | 0.24 | 0.31 |
| Leaf size [FE] | 10% | 10 | allele ratio | 0.3 | 0.28 | 0.29 |
| Leaf size [FE] | 10% | 20 | allele ratio | 0.3 | 0.32 | 0.22 |
| Leaf size [FE] | 10% | 30 | allele ratio | 0.3 | 0.27 | 0.29 |
| Leaf size [FE] | 10% | 40 | allele ratio | 0.28 | 0.26 | 0.3 |
| Leaf size [FE] | 20% | 10 | allele ratio | 0.29 | 0.3 | 0.27 |
| Leaf size [FE] | 20% | 20 | allele ratio | 0.3 | 0.3 | 0.29 |
| Leaf size [FE] | 20% | 30 | allele ratio | 0.29 | 0.31 | 0.25 |
| Leaf size [FE] | 20% | 40 | allele ratio | 0.24 | 0.23 | 0.25 |
| Dry Matter [FE] | 5% | 10 | tetraploid | 0.37 | 0.36 | 0.27 |
| Dry Matter [FE] | 5% | 10 | diploid | 0.31 | 0.35 | 0.25 |
| Dry Matter [FE] | 5% | 20 | tetraploid | 0.35 | 0.35 | 0.28 |
| Dry Matter [FE] | 5% | 20 | diploid | 0.31 | 0.33 | 0.23 |
| Dry Matter [FE] | 5% | 30 | tetraploid | 0.37 | 0.35 | 0.23 |
| Dry Matter [FE] | 5% | 30 | diploid | 0.33 | 0.34 | 0.22 |
| Dry Matter [FE] | 5% | 40 | tetraploid | 0.37 | 0.37 | 0.19 |
| Dry Matter [FE] | 5% | 40 | diploid | 0.33 | 0.36 | 0.17 |
| Dry Matter [FE] | 10% | 10 | tetraploid | 0.36 | 0.38 | 0.31 |
| Dry Matter [FE] | 10% | 10 | diploid | 0.39 | 0.36 | 0.32 |
| Dry Matter [FE] | 10% | 20 | tetraploid | 0.35 | 0.36 | 0.35 |
| Dry Matter [FE] | 10% | 20 | diploid | 0.36 | 0.37 | 0.29 |
| Dry Matter [FE] | 10% | 30 | tetraploid | 0.35 | 0.37 | 0.3 |
| Dry Matter [FE] | 10% | 30 | diploid | 0.35 | 0.37 | 0.23 |
| Dry Matter [FE] | 10% | 40 | tetraploid | 0.36 | 0.39 | 0.26 |
| Dry Matter [FE] | 10% | 40 | diploid | 0.35 | 0.37 | 0.24 |
| Dry Matter [FE] | 20% | 10 | tetraploid | 0.38 | 0.38 | 0.36 |
| Dry Matter [FE] | 20% | 10 | diploid | 0.37 | 0.4 | 0.33 |
| Dry Matter [FE] | 20% | 20 | tetraploid | 0.39 | 0.41 | 0.33 |
| Dry Matter [FE] | 20% | 20 | diploid | 0.37 | 0.38 | 0.34 |
| Dry Matter [FE] | 20% | 30 | tetraploid | 0.36 | 0.38 | 0.32 |
| Dry Matter [FE] | 20% | 30 | diploid | 0.35 | 0.36 | 0.29 |
| Dry Matter [FE] | 20% | 40 | tetraploid | 0.33 | 0.36 | 0.27 |
| Dry Matter [FE] | 20% | 40 | diploid | 0.35 | 0.35 | 0.25 |
| Dry Matter [FE] | 5% | 10 | allele ratio | 0.41 | 0.39 | 0.33 |
| Dry Matter [FE] | 5% | 20 | allele ratio | 0.39 | 0.36 | 0.3 |
| Dry Matter [FE] | 5% | 30 | allele ratio | 0.38 | 0.35 | 0.29 |
| Dry Matter [FE] | 5% | 40 | allele ratio | 0.35 | 0.33 | 0.27 |
| Dry Matter [FE] | 10% | 10 | allele ratio | 0.4 | 0.38 | 0.39 |
| Dry Matter [FE] | 10% | 20 | allele ratio | 0.35 | 0.37 | 0.37 |
| Dry Matter [FE] | 10% | 30 | allele ratio | 0.35 | 0.36 | 0.33 |
| Dry Matter [FE] | 10% | 40 | allele ratio | 0.33 | 0.36 | 0.33 |
| Dry Matter [FE] | 20% | 10 | allele ratio | 0.35 | 0.39 | 0.37 |
| Dry Matter [FE] | 20% | 20 | allele ratio | 0.37 | 0.36 | 0.36 |
| Dry Matter [FE] | 20% | 30 | allele ratio | 0.32 | 0.36 | 0.35 |
| Dry Matter [FE] | 20% | 40 | allele ratio | 0.35 | 0.37 | 0.35 |
| Dry Matter [SE] | 5% | 10 | tetraploid | 0.06 | 0.06 | 0.08 |
| Dry Matter [SE] | 5% | 10 | diploid | 0.1 | 0.06 | 0.1 |
| Dry Matter [SE] | 5% | 20 | tetraploid | 0.04 | 0.04 | 0.06 |
| Dry Matter [SE] | 5% | 20 | diploid | 0.12 | 0.07 | 0.16 |
| Dry Matter [SE] | 5% | 30 | tetraploid | 0.05 | 0 | 0.11 |
| Dry Matter [SE] | 5% | 30 | diploid | 0.05 | 0.05 | 0.17 |
| Dry Matter [SE] | 5% | 40 | tetraploid | 0.08 | 0.04 | 0.08 |
| Dry Matter [SE] | 5% | 40 | diploid | 0.08 | 0.08 | 0.1 |
| Dry Matter [SE] | 10% | 10 | tetraploid | 0.04 | 0.05 | 0.09 |
| Dry Matter [SE] | 10% | 10 | diploid | 0.09 | 0.07 | 0.1 |
| Dry Matter [SE] | 10% | 20 | tetraploid | 0.03 | 0.05 | 0.04 |
| Dry Matter [SE] | 10% | 20 | diploid | 0.08 | 0.09 | 0.06 |
| Dry Matter [SE] | 10% | 30 | tetraploid | 0.03 | 0.05 | 0 |
| Dry Matter [SE] | 10% | 30 | diploid | 0.06 | 0.09 | 0.05 |
| Dry Matter [SE] | 10% | 40 | tetraploid | 0.03 | 0.04 | 0.01 |
| Dry Matter [SE] | 10% | 40 | diploid | 0.06 | 0.07 | 0.08 |
| Dry Matter [SE] | 20% | 10 | tetraploid | 0.08 | 0.1 | 0.06 |
| Dry Matter [SE] | 20% | 10 | diploid | 0.09 | 0.12 | 0.05 |
| Dry Matter [SE] | 20% | 20 | tetraploid | 0.1 | 0.06 | 0.1 |
| Dry Matter [SE] | 20% | 20 | diploid | 0.06 | 0.1 | 0.12 |
| Dry Matter [SE] | 20% | 30 | tetraploid | 0.07 | 0.06 | 0.09 |
| Dry Matter [SE] | 20% | 30 | diploid | 0.09 | 0.12 | 0.12 |
| Dry Matter [SE] | 20% | 40 | tetraploid | 0.06 | 0.08 | 0.04 |
| Dry Matter [SE] | 20% | 40 | diploid | 0.12 | 0.12 | 0.06 |
| Dry Matter [SE] | 5% | 10 | allele ratio | 0.07 | 0.09 | 0.04 |
| Dry Matter [SE] | 5% | 20 | allele ratio | 0.07 | 0.11 | 0.02 |
| Dry Matter [SE] | 5% | 30 | allele ratio | 0.06 | 0.07 | 0.03 |
| Dry Matter [SE] | 5% | 40 | allele ratio | 0.1 | 0.09 | 0.04 |
| Dry Matter [SE] | 10% | 10 | allele ratio | 0.07 | 0.07 | 0.07 |
| Dry Matter [SE] | 10% | 20 | allele ratio | 0.08 | 0.09 | 0.03 |
| Dry Matter [SE] | 10% | 30 | allele ratio | 0.07 | 0.06 | 0.03 |
| Dry Matter [SE] | 10% | 40 | allele ratio | 0.09 | 0.08 | 0.07 |
| Dry Matter [SE] | 20% | 10 | allele ratio | 0.06 | 0.07 | 0.06 |
| Dry Matter [SE] | 20% | 20 | allele ratio | 0.06 | 0.06 | 0.07 |
| Dry Matter [SE] | 20% | 30 | allele ratio | 0.08 | 0.08 | 0.09 |
| Dry Matter [SE] | 20% | 40 | allele ratio | 0.03 | 0.07 | 0.05 |
| Onset of flowering [FE] | 5% | 10 | tetraploid | 0.13 | 0.09 | 0.23 |
| Onset of flowering [FE] | 5% | 10 | diploid | 0.11 | 0.09 | 0.14 |
| Onset of flowering [FE] | 5% | 20 | tetraploid | 0.14 | 0.07 | 0.27 |
| Onset of flowering [FE] | 5% | 20 | diploid | 0.13 | 0.07 | 0.16 |
| Onset of flowering [FE] | 5% | 30 | tetraploid | 0.16 | 0.09 | 0.26 |
| Onset of flowering [FE] | 5% | 30 | diploid | 0.14 | 0.09 | 0.13 |
| Onset of flowering [FE] | 5% | 40 | tetraploid | 0.15 | 0.09 | 0.19 |
| Onset of flowering [FE] | 5% | 40 | diploid | 0.09 | 0.09 | 0.08 |
| Onset of flowering [FE] | 10% | 10 | tetraploid | 0.16 | 0.16 | 0.2 |
| Onset of flowering [FE] | 10% | 10 | diploid | 0.09 | 0.06 | 0.14 |
| Onset of flowering [FE] | 10% | 20 | tetraploid | 0.19 | 0.2 | 0.24 |
| Onset of flowering [FE] | 10% | 20 | diploid | 0.15 | 0.1 | 0.15 |
| Onset of flowering [FE] | 10% | 30 | tetraploid | 0.22 | 0.17 | 0.25 |
| Onset of flowering [FE] | 10% | 30 | diploid | 0.14 | 0.12 | 0.21 |
| Onset of flowering [FE] | 10% | 40 | tetraploid | 0.26 | 0.21 | 0.3 |
| Onset of flowering [FE] | 10% | 40 | diploid | 0.17 | 0.08 | 0.24 |
| Onset of flowering [FE] | 20% | 10 | tetraploid | 0.23 | 0.2 | 0.23 |
| Onset of flowering [FE] | 20% | 10 | diploid | 0.15 | 0.1 | 0.18 |
| Onset of flowering [FE] | 20% | 20 | tetraploid | 0.21 | 0.19 | 0.2 |
| Onset of flowering [FE] | 20% | 20 | diploid | 0.15 | 0.16 | 0.16 |
| Onset of flowering [FE] | 20% | 30 | tetraploid | 0.23 | 0.22 | 0.22 |
| Onset of flowering [FE] | 20% | 30 | diploid | 0.17 | 0.17 | 0.15 |
| Onset of flowering [FE] | 20% | 40 | tetraploid | 0.24 | 0.21 | 0.22 |
| Onset of flowering [FE] | 20% | 40 | diploid | 0.18 | 0.18 | 0.17 |
| Onset of flowering [FE] | 5% | 10 | allele ratio | 0.25 | 0.22 | 0.21 |
| Onset of flowering [FE] | 5% | 20 | allele ratio | 0.21 | 0.2 | 0.25 |
| Onset of flowering [FE] | 5% | 30 | allele ratio | 0.25 | 0.18 | 0.29 |
| Onset of flowering [FE] | 5% | 40 | allele ratio | 0.23 | 0.15 | 0.3 |
| Onset of flowering [FE] | 10% | 10 | allele ratio | 0.21 | 0.2 | 0.21 |
| Onset of flowering [FE] | 10% | 20 | allele ratio | 0.25 | 0.21 | 0.22 |
| Onset of flowering [FE] | 10% | 30 | allele ratio | 0.23 | 0.19 | 0.25 |
| Onset of flowering [FE] | 10% | 40 | allele ratio | 0.24 | 0.16 | 0.29 |
| Onset of flowering [FE] | 20% | 10 | allele ratio | 0.22 | 0.2 | 0.19 |
| Onset of flowering [FE] | 20% | 20 | allele ratio | 0.23 | 0.25 | 0.2 |
| Onset of flowering [FE] | 20% | 30 | allele ratio | 0.23 | 0.23 | 0.21 |
| Onset of flowering [FE] | 20% | 40 | allele ratio | 0.24 | 0.2 | 0.24 |
